# Association Between Laboratory Coagulation Parameters and Postpartum Hemorrhage in Preterm and Term Caesarean Section: A Retrospective Analysis

**DOI:** 10.3390/jcm13216604

**Published:** 2024-11-03

**Authors:** Christoph Dibiasi, Emilia Jecel, Veronica Falcone, Eva Schaden, Johannes Gratz

**Affiliations:** 1 Department of Anaesthesia, Intensive Care Medicine and Pain Medicine, Medical University of Vienna, 1090 Vienna, Austria; 2 Ludwig Boltzmann Institute for Digital Health and Patient Safety, Medical University of Vienna, 1090 Vienna, Austria; 3 Department of Obstetrics and Gynecology, Medical University of Vienna, 1090 Vienna, Austria

**Keywords:** anemia, blood coagulation, postpartum hemorrhage, preterm birth

## Abstract

**Background:** Deranged antepartum laboratory parameters may be risk factors for postpartum hemorrhage (PPH). However, whether this is also valid in women who give birth prematurely is currently unknown. **Methods:** We performed a retrospective single-center study to assess the role of antepartum hemoglobin, platelet count, fibrinogen, activated partial thromboplastin time, and prothrombin time as risk factors for PPH following caesarean section. We defined PPH as documented blood loss of at least 1 L and/or transfusion of red blood cell concentrates. We stratified the included patients according to gestational age: extremely preterm (gestational age < 28 weeks), very preterm (gestational age between 28 and 32 weeks), late and moderate preterm (gestational age between 32 and 37 weeks), and term (gestational age ≥ 37 weeks). **Results:** We included 1734 patients, 112 (6%) of whom had PPH. In total, 19 patients (10%) were in the extremely preterm group, 13 patients (10%) were in the very preterm group, 44 patients (9%) were in the late and moderate preterm group, and 36 patients (4%) were in the term group. Hemoglobin predicted PPH in all gestational age groups. Platelet count was associated with PPH in term, but not in preterm patients. Fibrinogen was associated with PPH in late prematurity but not in term patients and not in patients with early or extreme prematurity. **Conclusions:** Antepartum hemoglobin was the only factor predicting PPH in preterm and term caesarean sections. Platelet count and fibrinogen concentration were associated with PPH in term and late prematurity, respectively, but not in earlier stages of prematurity.

## 1. Introduction

Hemostatic balance is shifted towards a procoagulant state in pregnancy: increased concentration and activity of procoagulant coagulation factors parallel decreases in anticoagulatory activity and fibrinolytic capacity [1,2]. This translates to an increased risk for venous thrombosis and pulmonary embolism during pregnancy compared to non-pregnant women [3]. The extent of hypercoagulability increases during pregnancy, and its peak is usually observed at term [4]. Despite these procoagulant adaptions, postpartum hemorrhage (PPH) is a common complication, both in vaginal birth, where it is defined as blood loss ≥ 500 mL [5], as well as in caesarean section (CS), where it is defined as blood loss ≥ 1 L [5]. PPH carries the risk of significant morbidity and mortality [6]. Multiple studies have found preoperatively determined laboratory coagulation parameters to be associated with PPH: Most importantly, hypofibrinogenemia increases the risk for PPH [7,8,9,10], and although cut-off values are difficult to define, it is generally accepted that fibrinogen concentration below 2 g/L aggravates PPH [11]. Anemia [12,13], thrombocytopenia [14], and hypocalcemia [15] are also independent risk factors for the development of PPH during vaginal delivery and CS.

Globally, about 11% of all births are premature [16], i.e., before 37 weeks of pregnancy, and this rate is rising [17]. Both preterm birth and CS increase the risk of PPH [18]. However, many studies that investigated the association of abnormal laboratory coagulation parameters and PPH either only included women at term [12], did not analyze the influence of gestational age (GA) [10] or did not report GA of study participants [13,19]. Thus, little is known about whether abnormal coagulation parameters carry the same weight in premature women as in parturients at term.

In this study, we aimed to describe coagulation parameters in patients who underwent CS at varying stages of prematurity and at term. Furthermore, we assessed the relationship between laboratory coagulation parameters (hemoglobin concentration, platelet count, fibrinogen concentration, activated partial thromboplastin time (aPTT), and prothrombin time (PT)) and the development of primary PPH for each stage of prematurity.

## 2. Materials and Methods

This was a single-center, retrospective, observational study performed at the Medical University of Vienna, Austria.

### 2.1. Patients and Data Export

We screened the electronic health records of all women who underwent surgery at the Department of Obstetrics and Gynecology of the Medical University of Vienna, Austria, from January 2015 to December 2019 for study eligibility. We included all CS patients who had laboratory parameters determined up to seven days prior to the date of CS. For all eligible patients, we exported the following data from the IntelliSpace Critical Care and Anaesthesia patient data management system (Philips Austria GmbH, Vienna, Austria): age, documented blood loss, administered red blood cell (RBC) transfusions, and laboratory parameters (hemoglobin concentration, platelet count, fibrinogen concentration, aPTT, and PT); all data were obtained within the seven days prior to CS. We excluded patients who were missing all laboratory parameters. Data were accessed on 9 November 2022. The authors could identify individual participants during data collection. The primary outcome of our study was the development of primary PPH, which we defined as (a) documented blood loss of at least 1 L and/or (b) documented administration of at least one RBC concentrate intraoperatively or in the following 24 h. Secondary outcomes included the individual components of the composite primary outcome. Exposure variables included hemoglobin concentration, fibrinogen concentration, aPTT, and PT. We stratified all study participants according to gestational age into four groups based on the World Health Organization’s definition of premature birth: extremely preterm for gestational age < 28 weeks, very preterm for gestational age between 28 and 32 weeks, late and moderate preterm (LAMP) for gestational age between 32 and 37 weeks, and term for gestational age ≥ 37 weeks [20]. We did not perform a sample size calculation for this study, as we analyzed all patients available in our database.

### 2.2. Statistical Analysis

We provide descriptive statistics of the primary and secondary outcomes stratified by gestational age group. We report continuous variables as median and interquartile range (IQR) and ordinal variables with absolute and relative frequencies. To analyze the effect of the exposure variables on the primary outcome PPH, we performed logistic regression with all laboratory parameters as independent variables and with the following adjusting variables (age, parity, previous CS, pre-eclampsia or eclampsia, abnormal placentation, premature detachment of the placenta, and bleeding prior to CS). We decided to adjust the variables prior to data analysis based on previously described risk factors for PPH and our clinical experience. We obtained odds ratios for the development of PPH together with 95% confidence intervals (CI) for each of the exposure variables.

Data analysis was performed using R version 4.2.2 (R Foundation for Statistical Computing, Vienna, Austria).

## 3. Results

**Figure 1:**
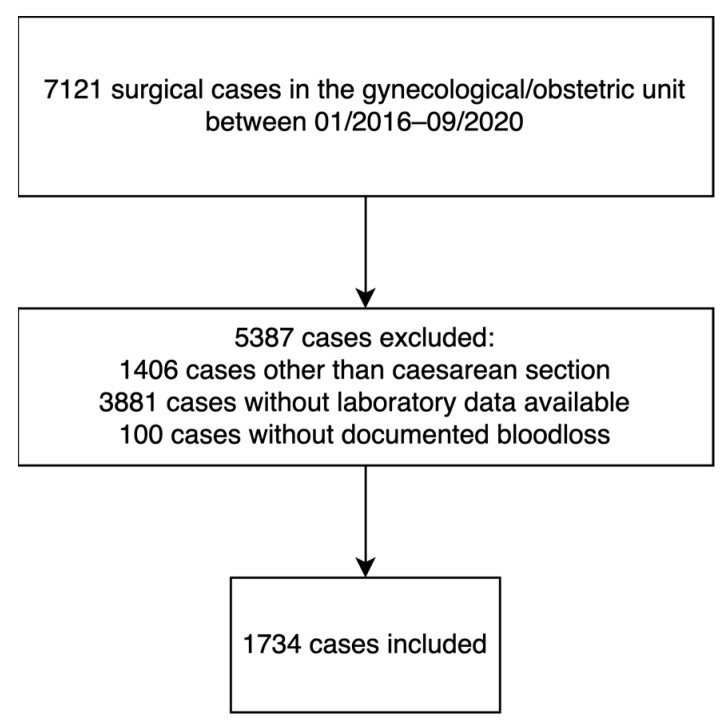
STROBE flowchart.

We included a total of 1734 patients (Figure 1) with an overall median (IQR) gestational age of 37 weeks (33–39). We grouped patients based on gestational age according to the WHO classification of premature birth into one of four groups: 188 (11%) women were in the extremely preterm birth group (i.e., gestational age up to 28 weeks), 136 (8%) were in the very preterm birth group (i.e., gestational age between 28 and 32 weeks), 481 (28%) were in the LAMP birth group (i.e., gestational age between 32 and 37 weeks), and 929 (54%) were in the term birth group (i.e., gestational age at least 37 weeks). Actual median (IQR) gestational age was 26 (25–27) weeks, 30 (29–31) weeks, 35 (33–36) weeks, and 39 (38–40) weeks, respectively, in the four groups. The baseline characteristics of the included patients are given in Table 1.

**Figure 2:**
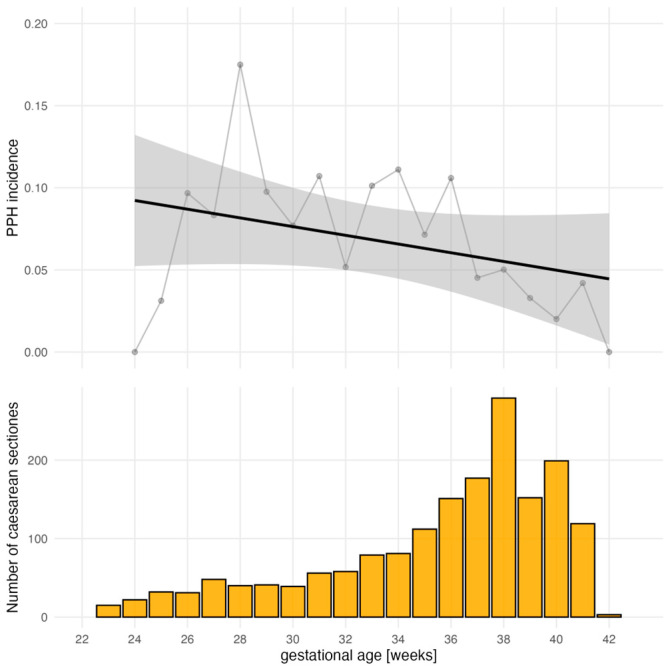
Incidence of postpartum hemorrhage decreases with gestational age.

A total of 112 (6%) patients developed PPH. The development of PPH differed significantly depending on the gestational age group (*p* < 0.001): 19 (10%) patients with PPH were in the extreme preterm group, 13 (10%) in the very preterm group, 44 (9%) in the LAMP group, and 36 (4%) patients with PPH were in the term group. The risk of PPH decreased with increasing gestational age by 7% (95% CI 4 to 11; *p* < 0.001) per additional week over gestational age of 23 weeks (Figure 2).

Preoperative laboratory parameters were slightly different across the four gestational age groups (Table 2). Hemoglobin and fibrinogen concentrations were increased among the term patients, whereas their platelet counts were slightly lower compared to patients in the extremely preterm and very preterm birth groups. PTT and PT did not change among the gestational age groups. We found that the preoperative hemoglobin concentration was lower in patients who developed PPH (for extremely preterm births, LAMP births, and in term births; Table 2). In addition, the preoperative fibrinogen concentration was lower in patients with PPH in LAMP births and term births (Table 2). Other laboratory parameters did not differ between patients developing PPH and patients without PPH (Table 2).

Analysis of the entire patient sample showed that hemoglobin and fibrinogen concentrations were risk factors for developing PPH (Table 3). Subgroup analysis of each gestational age group indicated that a low hemoglobin concentration increased the risk of PPH in extremely preterm births, LAMP births, and term births. Low fibrinogen concentration increased the risk of PPH only in the LAMP birth group. Platelet count was associated with PPH in term patients only but not in patients with preterm birth. We describe the association between laboratory parameters and the secondary outcomes (blood loss of at least 1 L, transfusion of at least one RBC concentrate) in Tables S1 and S2. Here, we found that preoperative hemoglobin concentration was associated with blood loss of at least 1 L and RBC concentrate administration in the analysis of the entire patient sample. Fibrinogen concentration was only associated with RBC concentrate administration.

We also analyzed the effect of patient’s body mass index (BMI) on laboratory parameters. We found that only fibrinogen concentration was correlated with BMI (r = 0.15, *p* < 0.001), while the other laboratory parameters (hemoglobin, platelet count, PTT, and PT) were not associated with BMI (hemoglobin: r = −0.04, *p* = 0.215; platelet count: r = 0.07, *p* = 0.024; PT: r = −0.03, *p* = 0.342, PT: r = −0.03, *p* = 0.300). BMI was also not correlated with PPH (odds ratio 0.98 [95% CI 0.94–1.02]) in univariate analysis.

## 4. Discussion

We showed that lower hemoglobin levels, platelet counts, and fibrinogen levels were associated with a higher risk for PPH in pregnant patients who underwent CS. The effects were more pronounced in patients with term and LAMP births. In the extremely preterm birth group, only the preoperative hemoglobin concentration remained a predictor of PPH. Standard laboratory coagulation times, that is, PTT and PT, were within normal limits and were not associated with the development of PPH in any of the preterm birth groups. Our results indicate that in patients with early gestational age, hemoglobin is the only preoperative laboratory parameter potentially useful for estimating the risk of PPH during CS.

Preoperative PPH risk assessment is an important task to appropriately decide on periprocedural management, to guide blood product allocation, and to counsel patients. Individualized risk prediction of PPH is difficult, and current guidelines from Germany, Austria, and Switzerland do not recommend a specific PPH prediction tool but rather advocate individualized assessments based on previously described risk factors [5]. Anemia is a well-known factor that increases the risk of PPH [12,13,21]. Previous studies have reported odds ratios for the development of PPH to be between 0.58 (95% CI 0.46–0.73) [13] and 0.86 (95% CI 0.78–0.90) [12] per 1 g/dl increase in hemoglobin in patients with term births. Our study confirms this relationship in term patients who underwent CS and furthermore shows that the association between anemia and PPH also holds true in patients with gestational age below 37 weeks. A possible underlying reason for this could be worse uterine contractability, as arterial oxygen content is reduced in anemia [22]. In addition, lower blood clot stiffness has been demonstrated in experimental anemia [23] and could, in theory, be responsible for worse hemostasis in anemic patients [24].

Mild thrombocytopenia is common during pregnancy [25]. Platelet counts below 1.5 G/L have been shown to increase the risk of PPH in term pregnancies [7,14,26].

Our analysis shows that low platelet counts even above the lower border of the reference range are associated with PPH in term patients but not in any of the three prematurity patient groups. Previous studies on the value of fibrinogen as a predictor of PPH have been conflicting, with some studies describing associations between low antepartum fibrinogen in healthy parturients [7,8,9,14] and patients with HELLP syndrome [19], while others have found only a weak [10] or no association at all [21,27,28]. We did not find an association between preoperative fibrinogen concentration and PPH undergoing CS in the term group, but there was an association in the late prematurity group. The diminished role of platelet count and fibrinogen concentration in earlier stages of pregnancy (i.e., in the extremely preterm and very preterm groups) could possibly be explained by the increased importance of other perioperative factors. Pre-eclampsia and/or eclampsia, as well as abnormal placentation or antepartum bleeding, were more common in patients in the preterm group who underwent CS and were by themselves associated with PPH. In addition, CS is technically more difficult prior to a gestational age of 32 weeks as the uterus is smaller and more thick-walled, which hinders child development and could be associated with higher blood loss.

Preoperative standard laboratory coagulation tests—that is, PT and PTT—have previously been described not to be useful as screening tests for PPH [7], which is further corroborated by our data. Therefore, the determination of standard laboratory tests should be reserved for patients with clinical bleeding abnormalities. Potential alternatives to standard laboratory assays are viscoelastic coagulation tests (VETs), which are recommended by current guidelines for the management of actively bleeding parturients [29]. VETs help clinicians to diagnose coagulopathy and can guide hemostatic treatment. The introduction of VETs in obstetrics has led to reductions in allogenic blood transfusion, ICU admission and hysterectomy [30,31]. However, VETs have so far failed to be useful antepartum markers of PPH [28]. Novel VETs feature additional tests, e.g., the tissue plasminogen activator test, which can be used to assess endogenous and exogenous inhibition of fibrinolysis. This “fibrinolytic potential” was associated with PPH in one study and could as such be potentially used as antepartum indicator for an increased risk of PPH [32].

Several limitations hinder the direct applicability of our study in clinical practice. First, we included only patients for whom laboratory parameters were electronically available, which introduces selection bias. In addition, we are unable to assess the contribution of coagulopathy such as van Willebrand syndrome or platelet disorders, as such data were not available to us. Second, blood loss was estimated visually by the surgeons after completion of the operation, which has been shown to be imprecise [33]. We therefore opted to use the composite of blood loss above 1 L and/or transfusion of at least one RBC concentrate as the primary outcome, as we consider RBC administration a clinically relevant event that—when performed during surgery—indicates relevant blood loss. Third, due to the missing sample size calculation and lack of hypothesis formulation, our results should be deemed exploratory and should be confirmed in future trials with a more stringent methodology.

In summary, we analyzed preoperatively determined laboratory parameters from 1734 women undergoing CS and found that hemoglobin was the only factor predicting PPH in all three stages of prematurity and in term birth. Although platelet count was associated with PPH in term and fibrinogen was associated with PPH in late prematurity patients, these associations did not hold true for patients in the early and extreme prematurity birth group. Based on our results, standard laboratory coagulation tests are not useful for estimating the risk of PPH.

## Figures and Tables

**Table 1:** Baseline characteristics of the study cohort.

	Extremely Preterm N = 188	Very Preterm N = 136	Late and Moderate Preterm N = 481	Term N = 929
**Age (years)**	32 (28–36)	32 (29–37)	34 (29–37)	32 (28–37)
**Weight (kg)**	76 (68–87)	74 (65–86)	77 (69–90)	80 (70–91)
**Height (kg)**	165 (162–170)	165 (160–168)	165 (160–170)	165 (160–170)
**Body mass index (kg/m^2^)**	27.6 (24.7–31.2)	26.6 (24–31.5)	28.2 (25–33.2)	29.1 (25.5–33.1)
**ASA Score (-)**				
1	84 (45)	59 (43)	195 (41)	452 (49)
2	71 (38)	49 (36)	227 (47)	407 (44)
3	26 (14)	23 (17)	51 (11)	48 (5.2)
4	4 (2.1)	1 (0.7)	3 (0.6)	1 (0.1)
5	3 (1.6)	4 (2.9)	5 (1.0)	20 (2.2)
**Urgency (-)**				
Elective	37 (20)	33 (24)	198 (41)	458 (49)
Urgent	73 (39)	68 (50)	194 (40)	277 (30)
Emergency	78 (41)	35 (26)	89 (19)	193 (21)
**PPH (%)**	19 (10)	13 (9.6)	44 (9.1)	36 (3.9)
**Estimated blood loss (mL)**	300 (300–450)	300 (300–438)	350 (300–500)	300 (300–400)
**Estimated blood loss ≥ 1 L (-)**	11 (5.9)	9 (6.7)	25 (5.2)	23 (2.5)
**Blood transfusion (-)**	12 (6.4)	9 (6.6)	36 (7.5)	21 (2.3)
**ICU admission (-)**	11 (5.9)	11 (8.1)	23 (4.8)	11 (1.2)
**Past pregnancies (-)**	2 (1–3)	2 (1–3)	2 (1–3)	2 (1–3)
**Parity (-)**				
1	150 (80)	86 (63)	340 (71)	874 (94)
2	34 (18)	45 (33)	133 (28)	54 (5.8)
3	4 (2.1)	5 (3.7)	8 (1.7)	1 (0.1)
**Previous CS (-)**	7 (3.7)	10 (7.4)	90 (19)	228 (25)
**Pre-eclampsia or eclampsia (-)**	35 (19)	23 (17)	79 (16)	33 (3.6)
**Abnormal placentation (-)**	8 (4.3)	6 (4.4)	31 (6.4)	12 (1.3)
**Bleeding prior to CS (-)**	6 (3.2)	2 (1.5)	5 (1.0)	13 (1.4)
**Placental abruption (-)**	7 (3.7)	9 (6.6)	11 (2.3)	4 (0.4)
**Bleeding diathesis (-)**	0 (0)	0 (0)	0 (0)	6 (0.6)

All data given as median and interquartile range or as absolute and relative counts. ASA: American Society of Anesthesiology; PPH: postpartum hemorrhage; ICU: intensive care unit; CS: caesarean section.

**Table 2:** Preoperatively determined laboratory parameters stratified by gestational age group and development of PPH.

	Extremely Preterm			Very Preterm			Late and Moderate Preterm			Term		
	No PPH N = 169	PPH N = 19	*p*	No PPH N = 123	PPH N = 13	*p*	No PPH N = 437	PPH N = 44	*p*	No PPH N = 893	PPH N = 36	*p*
**Hemoglobin (g/dL)**	**11.4 (10.4–12.2)**	**10.6 (9.5–11.2)**	**0.005**	11.50 (10.4–12.2)	11.4 (10.5–11.7)	0.65	**11.6 (10.7–12.6)**	**10.1 (9.0–11.1)**	**<0.001**	**11.9 (11.0–12.8)**	**11.5 (10.1–12.1)**	**0.010**
**Platelet count (100 G/L)**	2.38 (1.84–2.75)	2.21 (1.91–2.60)	0.49	2.22 (1.84–2.52)	2.22 (1.97–2.66)	>0.99	2.12 (1.72–2.48)	2.00 (1.51–2.42)	0.25	**2.21 (1.81–2.63)**	**1.99 (1.47–2.23)**	**0.040**
**Fibrinogen (g/L)**	4.59 (3.97–5.40)	4.60 (3.70–5.33)	0.62	4.86 (4.04–5.47)	4.34 (3.81–4.90)	0.18	**4.90 (4.32–5.46)**	**4.43 (3.58–4.90)**	**<0.001**	4.86 (4.31–5.43)	4.80 (4.01–4.95)	0.063
**Activated partial thromboplastin time (s)**	32.1 (30.2–33.9)	32.5 (30.6–33.5)	0.91	32.2 (30.2–34.4)	32.5 (30.8–34.5)	0.99	32.3 (30.7–34.0)	31.8 (30.1–33.3)	0.25	32.3 (30.7–34.1)	32.2 (30.9–33.0)	0.59
**Prothrombin time (s)**	24.6 (24.6–25.4)	24.6 (24.6–25.0)	0.57	**24.6 (24.6–24.6)**	**25.6 (24.6–26.5)**	**<0.001**	**24.6 (24.6–24.9)**	**24.6 (24.6–25.2)**	**0.032**	**24.6 (24.6–24.7)**	**24.6 (24.6–25.1)**	**0.025**

All data given as median with interquartile range. *p* values were obtained using Wilcoxon rank-sum tests. Bold values are statistically significant.

**Table 3:** Adjusted odds ratios for development of PPH for all patients and stratified by gestational age group.

	Overall			Extremely Preterm			Very Preterm			Late and Moderate Preterm			Term		
	OR	95% CI	*p*	OR	95% CI	*p*	OR	95% CI	*p*	OR	95% CI	*p*	OR	95% CI	*p*
**Hemoglobin (g/dL)**	**0.58**	**0.50–0.67**	**<0.001**	**0.51**	**0.32–0.77**	**0.002**	1.35	0.78–2.46	0.29	**0.47**	**0.36–0.61**	**<0.001**	**0.62**	**0.48–0.81**	**<0.001**
**Platelet count (G/L)**	0.77	0.56–1.05	0.11	1.31	0.49–3.26	0.57	1.61	0.37–7.79	0.54	0.97	0.53–1.73	0.92	**0.50**	**0.27–0.89**	**0.022**
**Fibrinogen (g/L)**	**0.76**	**0.59–0.98**	**0.034**	0.99	0.54–1.74	0.98	0.96	0.32–2.78	0.94	**0.48**	**0.29–0.78**	**0.004**	0.87	0.55–1.34	0.53
**Activated partial thromboplastin time (s)**	1.01	0.95–1.07	0.68	1.05	0.86–1.27	0.60	0.91	0.71–1.13	0.40	1.02	0.90–1.12	0.76	1.01	0.90–1.09	0.82
**Prothrombin time (s)**	1.06	0.89–1.24	0.49	0.60	0.26–1.07	0.14	**2.48**	**1.40–4.90**	**0.004**	0.92	0.59–1.33	0.70	1.10	0.83–1.38	0.45

OR = odds ratio; CI = confidence interval. Bold values are statistically significant.

## Data Availability

The dataset used in this study is available upon reasonable request.

## Supplementary Materials

The following supporting information can be downloaded at: https://www.mdpi.com/article/10.3390/jcm13216604/s1, Table S1: Adjusted odds ratios for documented blood loss of at least 1000 mL stratified by gestational age group, Table S2: Adjusted odds ratios for administration of at least one red blood cell concentrate stratified by gestational age group.
