# Supplementary material for: Association Between Laboratory Coagulation Parameters and Postpartum Hemorrhage in Preterm and Term Caesarean Section: A Retrospective Analysis
Source: J Clin Med. 2024 Nov 3;13(21):6604. doi: 10.3390/jcm13216604 (PMC11545883; doi:10.3390/jcm13216604)
Supplement: Supplementary file 1 [file jcm-13-06604-s001.zip › jcm-3207133-supplementary.pdf]

Association between laboratory coagulation parameters with postpartum haemorrhage in premature and at-term caesarean section: A retrospective analysis

Supplemental Material

Supplemental Table S1: Adjusted odds ratios for documented blood loss of at least 1000 mL stratified by gestational age group.

Supplemental Table S2: Adjusted odds ratios for administration of at least one red blood cell concentrate stratified by gestational age group.

Supplemental Table S1: Adjusted odds ratios for documented blood loss of at least 1000 mL stratified by gestational age group.

	**Overall**			**extremely preterm**			**very preterm**			**moderate to late preterm**			**at term**		
	OR	95% CI	p-value	OR	95% CI	p-value	OR	95% CI	p-value	OR	95% CI	p-value	OR	95% CI	p-value
**Haemoglobin (g/dL)**	0.74	0.61, 0.89	0.001	0.73	0.45, 1.17	0.19	0.90	0.43, 1.85	0.77	0.64	0.47, 0.87	0.005	0.81	0.57, 1.16	0.24
**Platelet count (G/L)**	0.81	0.53, 1.18	0.29	1.38	0.45, 3.80	0.54	0.89	0.11, 6.16	0.91	1.32	0.64, 2.66	0.45	0.38	0.16, 0.85	0.023
**Fibrinogen (g/L)**	0.78	0.56, 1.06	0.11	1.09	0.53, 2.06	0.79	1.16	0.33, 3.96	0.81	0.50	0.27, 0.89	0.022	0.80	0.43, 1.41	0.44
**Activated partial thromboplastin time (s)**	0.99	0.90, 1.06	0.74	0.98	0.77, 1.21	0.86	0.81	0.59, 1.05	0.13	1.05	0.93, 1.16	0.33	0.97	0.81, 1.09	0.70
**Prothrombin time (s)**	1.03	0.81, 1.26	0.80	0.98	0.45, 1.64	0.94	1.99	0.99, 4.13	0.047	0.77	0.39, 1.25	0.37	1.00	0.63, 1.36	>0.99

1 OR = Odds Ratio, CI = Confidence Interval

Supplemental Table S2: Adjusted odds ratios for administration of at least one red blood cell concentrate stratified by gestational age group.

	**Overall**			**extremely preterm**			**very preterm**			**moderate to late preterm**			**at term**		
	OR	95% CI	p-value	OR	95% CI	p-value	OR	95% CI	p-value	OR	95% CI	p-value	OR	95% CI	p-value
**Haemoglobin (g/dL)**	0.47	0.39, 0.56	<0.001	0.34	0.16, 0.61	<0.001	1.79	0.91, 4.24	0.12	0.40	0.28, 0.55	<0.001	0.44	0.31, 0.61	<0.001
**Platelet count (G/L)**	0.84	0.57, 1.22	0.38	1.56	0.37, 5.61	0.51	1.23	0.24, 6.98	0.80	0.99	0.50, 1.92	0.97	0.57	0.28, 1.10	0.10
**Fibrinogen (g/L)**	0.67	0.49, 0.91	0.011	0.79	0.32, 1.62	0.56	0.98	0.19, 5.28	0.98	0.37	0.20, 0.67	0.001	1.02	0.59, 1.74	0.93
**Activated partial thromboplastin time (s)**	1.03	0.96, 1.10	0.32	1.17	0.89, 1.51	0.25	1.06	0.75, 1.45	0.71	0.94	0.80, 1.08	0.42	1.04	0.92, 1.13	0.42
**Prothrombin time (s)**	1.04	0.85, 1.24	0.72	0.10	0.00, 0.65	0.10	2.48	1.24, 6.34	0.024	1.15	0.74, 1.66	0.48	1.09	0.76, 1.45	0.59

OR = Odds Ratio, CI = Confidence Interval
